# A penile fracture hidden behind a urethral bleeding

**DOI:** 10.1016/j.eucr.2023.102587

**Published:** 2023-10-16

**Authors:** Pedro Francisco Fernandes, Ana Marta Ferreira, Paulo Azinhais, Arnaldo Figueiredo

**Affiliations:** Department of Urology and Renal Transplantation, Centro Hospitalar e Universitário de Coimbra, Praceta Professor Mota Pinto, 3004-561, Coimbra, Portugal

**Keywords:** Penile fracture, Urethral injury

## Abstract

Penile fracture with urethral injury is uncommon. Diagnosis is usually based on clinical history and physical examination. Nonetheless, atypical presentation obliges complementary examinations to be performed.

We report a case of a 45-years-old man with urethral bleeding after a blunt penile trauma that was ultimately diagnosed as having a cavernous body laceration on top of an urethral rupture.

## 1 Introduction

Penile fracture is an uncommon urological emergency. It has an estimated incidence between 0.29 and 1.36 per 100,000, probably underestimated given the associated embarrassment in seeking medical attention.^1^ This condition is caused by blunt trauma of erect penis leading to tear of tunica albuginea.^2^^,^^3^ It typically happens when rigid penis strikes the partner's perineum or pubic bone during sexual intercourse. Forced flexion (taqaandan), masturbation, rolling over an erect penis during a morning tumescence, accidental falls are less common aetiologies.^2^^,^^4^^,^^5^

The audible crackle, pain and immediate detumescence are the classical hallmarks of a penile fracture. Characteristic signs also include swelling and ecchymosis (“aubergine” deformity).^2^^,^^4^^,^^6^ Difficulty or inability to voiding, blood in urethral meatus and haematuria are suggestive of urethral injury.^3^^,^^4^^,^^6^ Simultaneous urethral rupture is present between 6 to 38% of cases.^4^, ^5^, ^6^

We report an atypical presentation of a penile fracture with urethral injury without any classical symptoms or signs of cavernous bodies fracture. As far as we know, there is just one other case reporting a similar clinical picture, with urethrorrhagia being the unique presenting sign.^7^

## 2 Case presentation

A 45-years-old man presents to emergency department with urethral bleeding. The patient explained that it started during sexual intercourse after a blunt trauma of penis during vaginal penetration. The patient has denied hearing a cracking sound, pain, as well as immediate detumescence and swollen of penis. On physical examination there was traces of blood in the urethral meatus and a palpable small subcoronal spongious formation, which when compressed caused an increase of urethral bleeding. There was no evidence of penile swelling, changes of overlying skin, penile shaft tenderness or palpable defect in tunica albuginea.

**Fig. 1:**
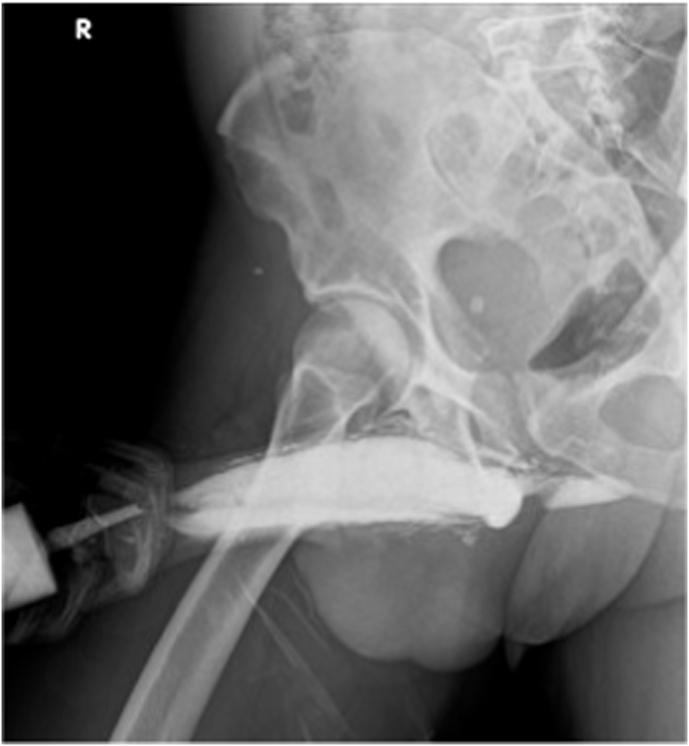
Retrograde urethrography with contrast only in anterior urethra and cavernous bodies.

A retrograde urethrography (RUG) was performed, which only showed an opacification of the anterior urethra and, in a very early phase, the filling of the cavernous bodies (Fig. 1). The penis ultrasound revealed an oval structure with 15 mm longer axis between cavernous and spongious bodies, with a liquid area inside. This lesion was compressible with the ultrasound probe but expanded as soon as the compression ceased.

**Fig. 2:**
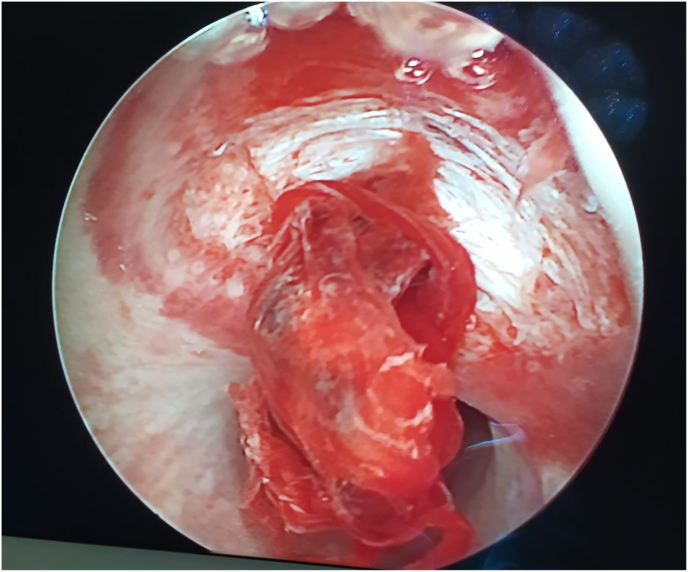
Urethroscopy showing a tear on dorsal face of the distal penile urethra.

A longitudinal tear on dorsal face of the distal penile urethra, extending approximately 1 cm, was observed on urethroscopy (Fig. 2). A 14 Fr catheter was inserted.

**Fig. 3:**
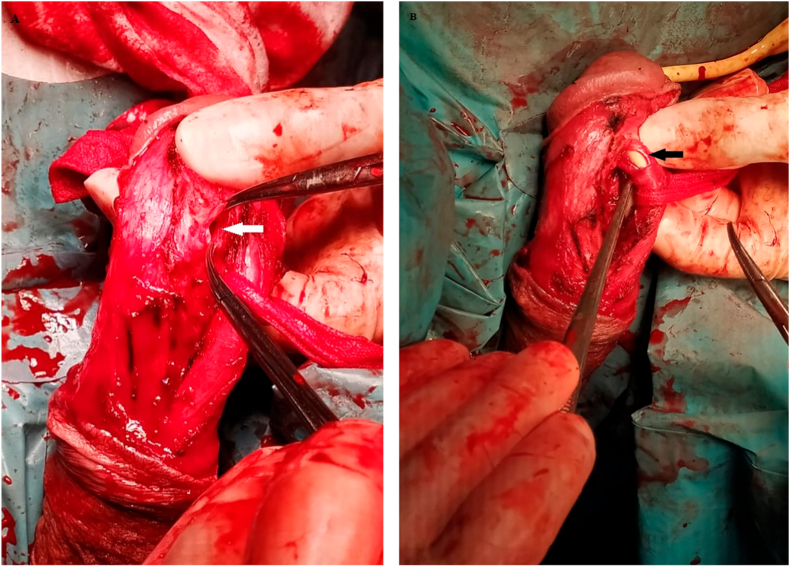
Intraoperative picture showing a hole of right *corpus cavernosum* (white arrow) (A) and laceration of penile urethra (black arrow) with Foley's catheter inserted (B).

No haematoma was identified after penile subcoronal degloving. Buck's fascia was opened parallel to the distal segment of *corpus spongiosum*, close to the site affected by the previously identified urethral disruption. A longitudinal laceration of the dorsolateral face of the urethra with approximately 1 cm was visualized. A rupture of the tunica albuginea of the juxtaposed right *corpus cavernosum* was also present (Fig. 3). The cavernous body lesion was sutured with polyglactin 0 and the urethral defect was closed in two planes, with interrupted polyglactin 4/0 sutures, first the mucosa and then the spongious body. The Buck's fascia was reconstituted with polyglactin 3/0. The patient was discharged on the following day and the urinary catheter was removed 14 days after the surgery.

Evaluation at one and six months revealed unchanged urinary flow (International Prostate Symptom Score (IPSS) questionnaire score was 0; bell-shaped uroflowmetry curve with a maximum flow rate of 21 mL/s) and normal and painless erections (6-month International Index of Erectile Function-5 (IIEF-5) questionnaire score was 25).

## 3 Discussion

Typical history and examination findings make the diagnosis of penile fracture predominantly clinical. Nonetheless, in our case there weren't any symptoms or signs indicating it - just a urethral bleeding after traumatic intercourse and a palpable small subcoronal spongious formation were present. Although extremely rare, isolated urethral injury without penile fracture at this setting (during sexual intercourse) has been described.^3^ Despite the disparities in epidemiology data (related to misreporting of penile fracture, among others), urethral bleeding and injury are documented in 5.6% patients with penile fracture.^1^^,^^5^

The early evaluation of male urethral injury comprises the performance of a RUG.^2^ In this case, it showed a contrast leakage from anterior urethra to cavernous body, revealing a penile fracture not perceived by clinical history nor by physical examination.

Flexible urethroscopy presents as an adjunct exam on urethral evaluation and it is preferred to RUG when the urethral injury is related to penile fracture, as RUG is associated with a high false-negative in these circumstances.^2^^,^^4^^,^^8^ Indeed, visual examination of the urethral mucosa allows to rule out or confirm urethral injury, its localization and extension, as well as to guide the insertion of a urethral catheter before surgical exploration.^2^^,^^5^^,^^8^ Of note, in our understanding this exam could be recommended if an urethral bleeding is present after a trauma whose mechanism predisposes to a penile fracture.

On doubtful cases, ultrasound and magnetic resonance imaging (MRI) can identify lacerations of tunica albuginea or provide reassurance that this structure is intact.^2^^,^^5^ MRI has been demonstrated to be very accurate^4^ and superior to ultrasound in diagnosing penile fracture,^2^ however its lower availability as well as the time required to perform it limit their use at emergency department.

In case of clinical suspicion of penile fracture, especially if it is associated with urethral disruption, surgical exploration should be done within 24 hours of presentation.^2^ Early surgical intervention is associated with significantly fewer complications than delayed or conservative management,^5^^,^^9^ the latter being associated with higher incidence of erectile dysfunction, plaques/nodules, penile curvature, painful erections and longer duration of inpatient stay and recovery.^5^ Primary urethroplasty in the context of penile fracture can lead to stenosis in 3% of cases.^4^ Some authors suggest separating the sutures of tunica albuginea repair and urethroplasty with a flap to avoid the formation of postoperative urethrocavernous fistula,^4^ although we did not to proceed with its placement due to the small extension of the defects identified. The duration of urethral catheterization depends also on the complexity of the repaired lesions, being 10 to 14 days or 14 to 21 days for partial or complete lesions, respectively.^4^

IPSS questionnaire and uroflowmetry are enough to evaluate postoperative urinary function. RUG is only recommended if there is any alteration on the aforementioned tools.^4^ IIEF-5 questionnaire and inquiry about the presence of penile curvature and pain on erection allow the assessment the sexual function.^9^

## 4 Conclusion

Blood in meatus is a clinical sign of urethral rupture when penile trauma is present. In these circumstances and even though the absence of symptoms and signs of *corpora cavernosa* fracture, a high index of suspicion must be maintained. Immediate exploration and reconstruction of urethra and tunica albuginea are indicated.

## Authors’ contributions

PFF wrote the initial draft and most significant edits of the manuscript. AMF, PA and AF reviewed and helped editing the manuscript. All authors read and approved the final submission.

## Funding

This work did not receive any specific grant from funding agencies in the public, commercial, or not-for-profit sectors.

## Availability of data and materials

Not applicable.

## Declaration of interests

The authors declare that they have no known competing financial interests or personal relationships that could have appeared to influence the work reported in this paper.

## Abbreviations

IIEF-5: International Index of Erectile Function-5
IPSS: International Prostate Symptom Score
MRI: magnetic resonance imaging
RUG: retrograde urethrography
